# Revisiting BPPV: Incidence and Behavior of Atypical Variants

**DOI:** 10.3390/audiolres15050140

**Published:** 2025-10-16

**Authors:** Salvatore Martellucci, Andrea Castellucci, Pasquale Malara, Luigi Califano, Giacinto Asprella Libonati

**Affiliations:** 1 ENT Unit, Santa Maria Goretti Hospital, AUSL Latina, 04100 Latina, Italy; dott.martellucci@gmail.com; 2 ENT Unit, Department of Surgery, Arcispedale Santa Maria Nuova, AUSL-IRCCS Reggio Emilia, 42123 Reggio Emilia, Italy; andrea.castellucci@ausl.re.it; 3 Audiology & Vestibology Service, Centromedico, 6500 Bellinzona, Switzerland; pasmalara@gmail.com; 4 Department of Audiology and Phoniatrics, San Pio Hospital, 82100 Benevento, Italy; luigi.califano1958@gmail.com; 5 ENT Unit, Santa Maria delle Grazie Hospital, ASM Matera, 75100 Matera, Italy

**Keywords:** BPPV, canalith jam, minimum stimulus strategy, atypical BPPV, canalolithiasis, cupulolithiasis, heavy cupula, down-beating nystagmus, video head impulse test, VHIT

## Abstract

**Objectives**: Typical BPPV forms are widespread and easily diagnosed disorders. However, some forms of labyrinthine lithiasis can differ from the typical BPPV paradigm, showing their own signs and symptoms and resulting in variable therapeutic responses. The aim of this retrospective study is to describe the incidence of the so-called atypical forms compared to the more common BPPV, describing their clinical behavior. **Methods**: This retrospective study analyzed clinical and instrumental data of 139 patients evaluated over a 12-month period at a referral center. Patients were divided into two groups. The first group (Group A) included patients with so-called “typical” and unilateral labyrintholithiasis, while the second group (Group B) included patients with so-called “atypical” forms. **Results**: Based on clinical characteristics, 82 patients were assigned to group A while 57 (51.01%) to group B. In group A, resolution of the clinical picture required fewer sessions and a smaller number of therapeutic maneuvers than in group B (*p* < 0.001). Furthermore, in group A, resolution of symptoms was observed immediately after one of the therapeutic maneuvers performed in 74.07% of cases, while in group B, resolution of the clinical picture was observed during one of the follow-up visits in 39.66% of cases (*p* < 0.001). **Conclusions**: Although considered rare, “atypical” forms have an increased prevalence in tertiary centers. The location of the canaliths within the labyrinth can be hypothesized based on the pattern of nystagmus, which serves as a guide for treatment.

## 1. Introduction

Benign Paroxysmal Positional Vertigo (BPPV) is the most common cause of peripheral vestibular vertigo, producing short-lasting but often disabling episodes of vertigo and nystagmus triggered by changes in head position relative to gravity [1,2,3]. While the majority of cases are idiopathic, BPPV may also result from head trauma, skull base or maxillofacial surgery, and inner ear disorders such as vestibular neuritis or Ménière’s disease [4,5,6,7]. Increasing evidence further links oxidative stress [8,9] and disturbances in calcium metabolism to the detachment of otoconia, with vitamin D deficiency emerging as a particularly relevant factor in patients with recurrent BPPV [10,11,12].

The widely accepted “lithiasis theory” explains BPPV as a mechanical disorder—essentially a “maculo-canalopathy”—in which otoconial debris, dislodged from the utricular macula, migrates into one or more semicircular canals (SCs). The resulting presence of these fragments within a canal leads to inappropriate stimulation of the ampullary receptor by gravitational forces [1,13]. Two main pathophysiological mechanisms have been described: canalolithiasis, in which free-floating debris within the canal lumen acts like a piston that drives endolymph and deflects the cupula, producing transient spells of vertigo with paroxysmal nystagmus; and cupulolithiasis, in which debris adheres to the cupula, rendering it gravity-sensitive and causing longer-lasting positional symptoms with non-paroxysmal nystagmus [1,3,13,14,15,16].

The diagnosis of BPPV typically requires identifying the affected ear, the involved canal, and the specific portion of the canal where the otoconial debris is localized. For this purpose, the most effective strategy combines a detailed patient history with a battery of positional tests designed to elicit transient positional vertigo accompanied by characteristic nystagmus. The Dix-Hallpike test (DHT), Semont, and Pagnini-McClure maneuvers are widely used to differentiate and diagnose typical BPPV variants [2,17,18,19].

According to the Bárány Society’s 2015 diagnostic criteria [19], three classical variants of BPPV are defined: posterior canal canalolithiasis, horizontal canal canalolithiasis (presenting with geotropic horizontal nystagmus), and horizontal canal cupulolithiasis (presenting with apogeotropic horizontal nystagmus). With respect to the latter variant, the guidelines explicitly note that apogeotropic direction-changing positional nystagmus is not pathognomonic of cupulolithiasis, since it may also result from horizontal canal canalolithiasis. Within this framework, the “typical” forms of BPPV as defined by the Bárány Society can therefore be categorized as:

- *Posterior canal canalolithiasis* (pc-BPPV);
- *Horizontal canal BPPV, geotropic form* (hc-BPPV-geo);
- *Horizontal canal BPPV, apogeotropic form* (hc-BPPV-apo).

The current guidelines of the American Academy of Otolaryngology—Head and Neck Surgery (2008, updated 2017) provide strong evidence-based recommendations for the diagnosis and treatment of these typical forms, emphasizing simple, non-invasive therapeutic maneuvers aimed at clearing the canal of displaced otoconia [17].

Yet, clinical reality is often less straightforward. Not all cases of positional vertigo neatly conform to these categories, nor do they always respond predictably to standard maneuvers. The Bárány Society itself highlights the existence of “rare” and “controversial” variants [19], among them:

- *Anterior canal canalolithiasis* (ac-BPPV), due to debris within the superior semicircular canal, leading to down-beating nystagmus (DBNy) at the DHT [20,21];
- *Posterior canal cupulolithiasis* (pc-BPPV-cu), due to otoconia are adherent to the cupula causing non-paroxysmal nystagmus upon provocation through the so-called “Half Dix-Hallpike Test” (HHT) [22,23,24,25];
- *Multi-canal lithiasis* (mc-BPPV), often following trauma or degeneration, in which debris affects more than one canal [26,27].

Over time, clinicians have also identified additional syndromes—both positional and non-positional—whose nystagmic patterns suggest labyrinthine lithiasis as the underlying pathophysiological mechanism, although they are not encompassed by traditional theories. Among these are:

- *Apogeotropic posterior canal canalolithiasis* (apo-pc-BPPV): characterized by DBNy during the DHT, resulting from debris lodged near the common crus [25,28,29,30,31,32,33,34,35];
- *Posterior canal BPPV with “sitting-up vertigo”* (pc-BPPV-suv): vertigo and nystagmus emerge as the patient returns to the upright position [36,37,38];
- *Canalith jam* (CJ), in which otoconia obstruct the narrowest portion of a canal, leading to persistent non-positional nystagmus [39,40,41,42,43,44], usually in the horizontal canal but occasionally involving vertical canals as well [45,46].

Based on the considerations above, in this study, we defined as “atypical BPPV” all lithiasic disorders not included in the Bárány Society guidelines, or those considered therein as “rare” or “controversial” variants. Diagnosing atypical BPPV requires more than routine maneuvers. Precise identification of the affected canal and the location of debris relies on a careful interpretation of nystagmus patterns evoked by head movements, rather than solely on a maneuver deemed “positive” simply because it provokes vertigo and an ill-defined nystagmus. Indeed, a DHT intended to confirm posterior canal involvement may, for example, unexpectedly elicit a nystagmic response indicative of another canal [47,48,49,50]. Beyond meticulous nystagmus analysis, the diagnosis of atypical forms can also be supported by complementary instrumental tests such as the video Head Impulse Test (vHIT), which quantifies vestibulo-ocular reflex (VOR) gain and can detect canal hypofunction caused by obstructive debris, thereby adding significant diagnostic value [42,45,46,51,52,53,54,55]. In atypical variants affecting the vertical canals, vHIT may be particularly useful in clarifying the site of pathology and localizing the otoconial mass [42,45,46,51,52,53,54,55].

Given the central role of nystagmus analysis, the “Minimum Stimulus Strategy” (MSS) has been introduced as a diagnostic model, providing a structured and standardized assessment through video-Frenzel monitoring of nystagmus while the head is gradually moved along different spatial axes [56,57,58,59,60]. A more recent development within this framework is the Upright BPPV Protocol (UBP), in which testing is carried out with the patient seated, relying on gravity to displace otoconia within the canals [60,61,62,63]. Originally designed to simplify the diagnosis of horizontal canal BPPV, UBP may also yield valuable insights into atypical forms.

Despite advances in knowledge and technology, atypical BPPV remains diagnostically and therapeutically challenging. A key gap persists regarding both the true incidence of these variants and their clinical course. This study therefore aims to address that gap by reporting the incidence of atypical forms relative to typical BPPV in a secondary referral center, describing their characteristic nystagmus during standard maneuvers including UBP, and assessing therapeutic outcomes with the aid of advanced tools such as vHIT.

## 2. Materials and Methods

This retrospective study reviewed records of patients with BPPV treated at a single secondary referral center from January 2023 to January 2024.

Exclusion criteria were: age < 18 years, incomplete medical records, and failure to adhere to the planned follow-up protocol after the initial visit (control examination within 5 days if symptoms and nystagmus persisted, followed by reassessments every 7 days until symptom resolution).

Patients requiring more than five evaluations were also excluded. A subset of patients experienced an “early recurrence,” defined as a new episode of positional vertigo on the same side occurring within 30 days after resolution of the previous episode. In such cases, only data from the first episode were considered for analysis.

### 2.1. Study Design

Clinical data collected included demographic information, time from symptom onset, descriptions of nystagmus observed during each diagnostic and therapeutic step, and the final diagnosis. When uncertainty arose, video recordings of the examination were reviewed. In addition, available vHIT outcomes were analyzed.

Patients were retrospectively classified into two groups: Group A included patients with “typical” unilateral BPPV, whereas Group B comprised those with “atypical” forms. The specific BPPV subtypes assigned to each group are summarized in Table 1. The main clinical characteristics associated with each diagnosis are summarized in Table 2 and Table 3.

All data were systematically entered into a Microsoft Excel spreadsheet (Microsoft Office 2024, Microsoft Corporation, Redmond, WA, USA) and processed for statistical analysis.

### 2.2. Examination Protocol and Diagnostic Criteria

**Figure 1:**
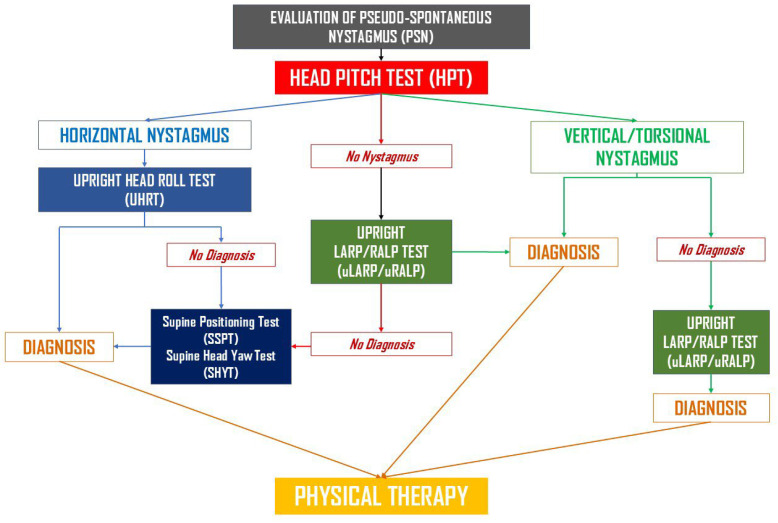
Flowchart illustrating the Upright BPPV Protocol (UBP) algorithm.

All patients were examined by the same experienced neurotologist (MS) using binocular video-Frenzel goggles (Nystalyze, Inventis S.R.L., Padua, Italy) following the standard protocol of the referral center. After a brief bedside evaluation—including assessment of ocular alignment, spontaneous and gaze-evoked nystagmus, saccades, and smooth pursuit—the diagnostic work-up proceeded as follows:

1. *Upright protocol (UBP):* The Upright BPPV Protocol consists of a battery of diagnostic tests performed in the sitting position, with the head moved along different spatial axes to displace canaliths by gravity within the involved canals. These include the Upright Head Pitch Test (uHPT), the Upright Head Roll Test (uHRT), and the Upright LARP/RALP test (uLARP/RALP). A detailed description of UBP can be found in the corresponding publications [60,61,62,63]. If UBP yielded a conclusive diagnosis, appropriate therapeutic maneuvers were immediately performed. A schematic representation of the Upright BPPV Protocol is shown in Figure 1. This flowchart summarizes the sequence of upright and traditional positional tests used to identify BPPV variants.
2. *Rapid positioning maneuvers (if Step 1 was non-diagnostic):* First, the Seated-to-Supine Positioning Test (SSPT), also known as the “Asprella Single Manoeuvre,” was performed [47,57,58,61,62]. If this was non-diagnostic, or if a vertical canal was suspected, the DHT was conducted. Conversely, if horizontal nystagmus was detected and lateral canal involvement was suspected, the Supine Head Yaw Test (sHYT)—also known as the Pagnini–McClure maneuver or the Supine Head Roll Test—was carried out. Traditional diagnostic maneuvers were performed in accordance with established clinical guidelines [17]. If the combination of UBP and classical positioning tests failed to establish a definite diagnosis, ancillary diagnostic procedures were employed, including the HHT (performed with the head slightly elevated from supine, approximately 30° in flexion), the Rose positioning test (supine with head and neck hyperextended), or the video Head Impulse Test (vHIT, ICS video-oculographic system, GN Otometrics, Ballerup, Denmark). The latter was used to identify the affected canal in cases of positional DBNy and in canalith jam conditions, as reported in previous studies [42,45,46,51,52,53].

Diagnostic criteria used in this study are summarized in Table 2 and Table 3. They were derived from the 2015 Consensus Document of the Committee for the Classification of Vestibular Disorders of the Bárány Society [19], and further integrated with data from the literature and the authors’ clinical experience. These criteria were based exclusively on the characteristics of nystagmus (direction, intensity, duration, and latency) and its variations during head positioning along different spatial planes.

According to the center’s protocol, patients underwent the therapeutic maneuver immediately after diagnosis and were re-evaluated after about 20 min. Those whose symptoms resolved were discharged, whereas those with persistent or modified BPPV received up to three additional maneuvers. If nystagmus and vertigo persisted, a follow-up was scheduled within 3–5 days, with further reassessments at the same interval until resolution. In cases of recurrence, patients were re-evaluated within 3–5 days.

### 2.3. Statistical Analysis

Continuous variables were summarized as mean, median, and standard deviation, while categorical variables were expressed as frequencies and percentages.

Associations between qualitative variables were tested using Pearson’s chi-square test for contingency tables. After verifying normal distribution, the independent samples *t*-test was used to compare two means. Statistical significance was set at *p* < 0.05. Statistical analyses were performed using the Statistical Package for the Social Sciences (SPSS), version 22.0 (IBM Corp., Armonk, NY, USA).

## 3. Results

A total of 139 patients were enrolled (82 females, 57 males; mean age 58 ± 15.7 years). The right ear was affected in 65 cases (46.76%), the left ear in 56 (40.29%), and bilateral involvement was observed in 11 cases (7.91%). In 7 patients (5.04%) the affected side could not be determined.

Based on clinical characteristics, 81 patients (58.27%) were classified into Group A (typical forms), while 58 (41.73%) were classified into Group B (atypical forms). The subgroup distribution is shown in Table 4. Of note, the group of patients with mc-BPPV could be further subdivided: 91.7% (11/12) had bilateral pc-BPPV, while 8.3% (1/12) had unilateral involvement (pc-BPPV with hc-BPPV-geo).

Regarding symptom onset (diagnostic delay), 40 patients (28.8%) presented within 48 h (hyperacute phase), 68 (48.9%) between 48 h and 7 days (acute phase), and 31 (22.3%) more than one week before evaluation (subacute phase). No significant differences were observed between the two groups in terms of age, sex, affected side, or diagnostic delay.

Head trauma or cranial surgery was reported in 1/81 patients in Group A (1.2%) and 2/58 in Group B (3.45%), with no significant difference between groups. A second vestibulopathy, either subacute, chronic, or recurrent, was observed in 4/81 patients in Group A (4.9%) and 11/58 in Group B (19%), representing a significant difference (*p* = 0.012).

The diagnosis was obtained exclusively through the UBP in 57 total cases (57/139, 41%). In particular, UBP was diagnostic in 55/81 patients from Group A (67.9%) and in 2/58 patients from Group B (3.4%). In Group A, this included 34/57 pc-BPPV cases (59.6%), 10/12 hc-BPPV-geo (83.3%), and 11/12 hc-BPPV-apo (91.7%). In Group B, both patients had mc-BPPV with bilateral involvement of the posterior semicircular canal.

vHIT was employed in 30 patients (30/139, 21.6%), including 28 of the 34 patients with BPPV associated with positional DBNy. In 16 of these 28 cases (57.1%), side and canal identification was supported by vHIT, which revealed a selectively reduced VOR gain (mean: 0.58 ± 0.07). Impaired VOR gain from the affected canal was also detected in both cases of canalith jam.

In Group A, pc-BPPV was treated with the Canalith Repositioning Procedure (CRP) according to Epley in 77.2% (44/57), the Semont maneuver in 8.8% (5/57), and combined approaches in 14.0% (8/57), including 2 cases of canal switch from the posterior to the horizontal canal [64]. Resolution occurred after one visit in 71.9% (41/57) and after two visits in 24.6% (14/57). In hc-BPPV-geo, 83.3% (10/12) received the Gufoni maneuver and 16.7% (2/12) combined approaches, with resolution after one visit in 66.7% (8/12) and after two visits in 33.3% (4/12). In hc-BPPV-apo, 41.7% (5/12) underwent the Zuma maneuver [65] and 58.3% (7/12) combined approaches, with resolution after one visit in 33.3% (4/12) and after two visits in 50.0% (6/12). Two patients (16.7%) required 3 or more visits.

In Group B, findings were more heterogeneous.

In the largest subgroup, patients with apo-pc-BPPV (*n* = 25), 52.0% (13/25) were treated exclusively with the Demi Semont maneuver [31], while the remaining 48.0% (12/25) required combined therapeutic strategies. Resolution was obtained in a single visit in 12.0% (3/25) of cases, in two visits in 36.0% (9/25), whereas 52.0% (13/25) required three or more visits. In ac-BPPV (*n* = 2), both patients were treated with the association of the liberatory maneuvers proposed by Yacovino [66] and Li [67], with resolution within two visits. In DBNy-BPPV-uc (*n* = 7), since the affected side could not be identified, treatment consisted of repeated liberatory or conversion attempts through a combination of maneuvers, including bilateral Demi Semont, repeated Li maneuvers, head shaking in hyperextension, and home dispersion maneuvers (Brandt–Daroff exercises). Resolution required three visits in 57.1% (4/7) and four to five visits in the remaining 42.9% (3/7).

In pc-BPPV-cu (*n* = 5), the “Bascule Maneuver” was attempted as first-line therapy. Resolution was obtained in a single visit in 40.0% (2/5), while the remaining 60.0% (3/5) required more than three visits. In pc-BPPV-suv (*n* = 5), one patient (20.0%) was resolved in a single visit, two (40.0%) in two visits, and the remaining two (40.0%) in more than two visits; in all cases, multiple therapeutic strategies were required.

In canalith jam (*n* = 2), several therapeutic attempts including head shaking and skull vibration were performed; vertigo resolved within two visits in one case, while four visits were necessary in the other.

Finally, among mc-BPPV patients (*n* = 12), all bilateral pc-BPPV cases (*n* = 11) were managed exclusively with the CRP, whereas the single patient with ipsilateral involvement of both posterior and horizontal canals underwent a combination of the CRP and Gufoni maneuvers. Resolution was obtained in a single visit in 50.0% (6/12), and no patient required more than three visits.

**Figure 2:**
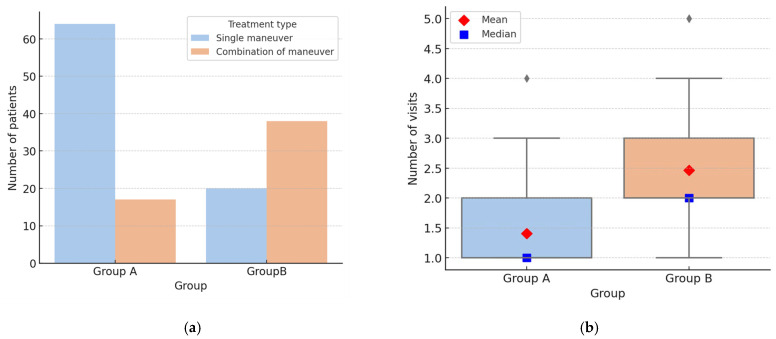
(**a**) Therapeutic strategies in Group A and Group B. (**b**) Box-plot illustrating the distribution of the number of therapeutic sessions required for symptom resolution in Group A and Group B.

From a therapeutic standpoint, in Group A, it was necessary to combine multiple therapeutic maneuvers in 17/81 cases (20.99%), whereas in Group B, the combination of different strategies was required in 38/58 cases (65.52%) (*p* < 0.001) (Figure 2a). Furthermore, a statistically significant difference between the two groups emerged when analyzing the number of visits required for complete clinical resolution: in typical forms, resolution was achieved after a mean of 1.41 ± 0.68 visits, compared with 2.47 ± 1.2 in atypical forms (*p* < 0.001) (Figure 2b).

Analysis of symptom resolution patterns revealed four paradigms: (a) immediate disappearance of vertigo and nystagmus; (b) conversion into an ipsilateral typical BPPV during the same visit; (c) disappearance of symptoms and nystagmus at the subsequent visit; (d) conversion into a typical BPPV at the subsequent visit. In this study, the term “conversion into an ipsilateral typical BPPV” was used to describe one of the observed resolution patterns, in which the nystagmus changes during repositioning maneuvers as a result of otoconial migration either within different segments of the same canal or into another canal whose nystagmus pattern corresponds to one of the recognized typical forms of BPPV. This definition applies to both Group A and Group B cases, as conversion phenomena may occur in typical as well as atypical variants before complete resolution.

Analysis of resolution paradigms revealed a difference between the two groups. Comparison of resolution patterns between the two groups revealed a significant difference (χ^2^ = 31.7, *p* < 0.000001): Group A was characterized by a predominance of immediate resolution, whereas Group B more frequently required later assessments or showed conversion into typical forms. The distribution of resolution paradigms in Group A and Group B is reported in Table 5.

In Group A, the majority of patients achieved immediate disappearance of vertigo and nystagmus (74.1%), whereas conversion during the same visit or resolution at a later assessment were infrequent (4.9% and 17.3%, respectively). In detail, 2 out of 57 (3.51%) cases of pc-BPPV underwent a canal switch, transforming into hc-BPPV during the same session and subsequently achieving immediate symptom resolution. In addition, 5 out of 12 (41.67%) cases of hc-BPPV-apo converted into the corresponding geotropic form (hc-BPPV-geo), two during the initial evaluation and the remaining three during follow-up visits.

**Figure 3:**
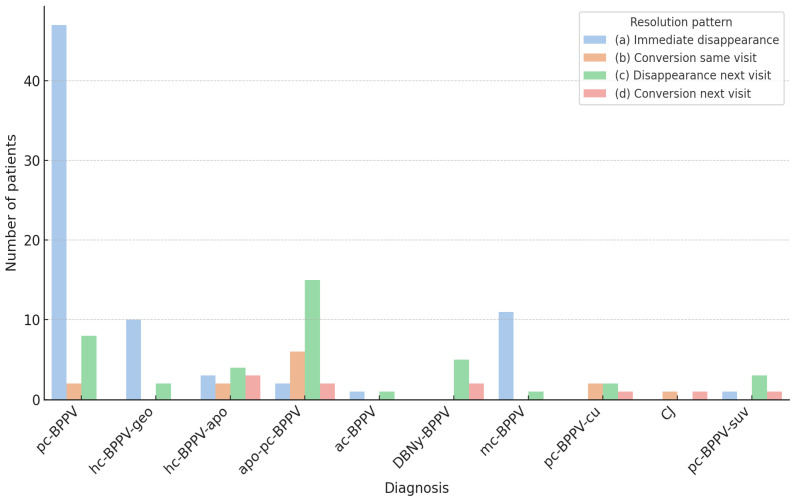
Distribution of resolution paradigms according to diagnosis. pc-BPPV, posterior canal canalolithiasis; hc-BPPV-geo, horizontal canal BPPV, geotropic form; hc-BPPV-apo, horizontal canal BPPV, apogeotropic form. apo-pc-BPPV, apogeotropic posterior canal canalolithiasis; mc-BPPV, multi-canal lithiasis; DBNy-BPPV, BPPV with downbeat ny, unspecified canal; pc-BPPV-suv, posterior canal BPPV with sitting-up vertigo; pc-BPPV-cu, posterior canal cupulolithiasis; CJ, Canalith Jam; ac-BPPV, anterior canal canalolithiasis.

In contrast, Group B showed a lower rate of immediate resolution (25.9%), largely due to mc-BPPV cases (11/15, 73.3%), and a higher frequency of patients requiring later assessments (46.6%) or showing conversion into typical forms (15.5% during the same visit and 12.1% at a later assessment). The distribution of resolution paradigms by diagnosis is illustrated in Figure 3. In detail, 8 out of 25 (32%) apo-pc-BPPV cases converted into the typical pc-BPPV form. In six cases, the conversion occurred during the examination and was directly observed by the examiner. Conversely, in the remaining two cases, the transformation into the typical form was detected at the subsequent visit. Likewise, in two cases of DBNy-BPPV (2/7, 28.57%), a typical pc-BPPV was diagnosed at a follow-up assessment and, according to the diagnostic criteria adopted in this study, both were classified as “conversion.” Conversion into a typical form was also documented in 3 out of 5 (60%) pc-BPPV-cu cases. In the two instances where the conversion was directly observed, the transformation resulted in hc-BPPV-geo, whereas transformation into pc-BPPV was observed in the single case of delayed conversion. Finally, transformation into pc-BPPV was also noted in one case of pc-BPPV-suv (1/5, 20%). Both cases of canalith jam (CJ) converted into hc-BPPV-apo and subsequently resolved following an additional conversion into the geotropic form (hc-BPPV-geo).

## 4. Discussion

Benign paroxysmal positional vertigo (BPPV) is one of the most frequent vestibular disorders encountered not only by otolaryngologists and neurologists, but also by general practitioners and emergency physicians [68,69]. About 60% to 80% of persons with BPPV consult primary care and hospital emergency departments [70].

Given its high prevalence and the fact that diagnosis is essentially clinical, without the need for specific instrumentation, most physicians can recognize and treat the commonest variants—the so-called ‘typical forms’ included in international guidelines [17,18,19,71], and even described in undergraduate medical textbooks and widely disseminated in online educational videos: pc-BPPV, hc-BPPV-geo, and hc-BPPV-apo. It should be noted, however, that some studies indicate that the CRP is less effective when performed by family physicians rather than experts [72].

Our data confirm what is already well established in the literature: treatment of these typical forms, particularly those involving the posterior canal (pc-BPPV) and the non-ampullary arm of the horizontal canal (hc-BPPV-geo), is highly effective. When canalolithiasis is the underlying mechanism, a single liberatory maneuver often leads to immediate resolution, and complications are rare. Several maneuvers have been validated, and their efficacy is generally comparable [73,74,75,76]. Hence, the choice of maneuver in daily practice depends largely on the examiner’s experience and preferences. For horizontal canal involvement, we favored the Gufoni and Zuma maneuvers, as their application directly following a diagnostic UBP—whose high diagnostic yield was confirmed in this cohort [77]—proved both practical for the examiner and more comfortable for patients, who remain seated throughout.

In the case of pc-BPPV, we propose that the CRP—by virtue of its slower execution and reliance on gravitational forces—allows for more reliable monitoring of nystagmus, thereby facilitating a clearer interpretation of otoconial mass dynamics within the labyrinth. Nonetheless, in patients with limited cervical range of motion, the efficacy of this maneuver may be compromised [78].

Among the 57 patients with pc-BPPV in our series (representing over 40% of the entire cohort), an immediate canal switch was observed in only two cases. This phenomenon, in which canaliths dislodged from the posterior canal migrate into the non-ampullary arm of another canal [64,79], has been reported to occur more frequently during the CRP than with the Semont maneuver. In our cohort, both switches involved the posterior-to-horizontal transition and were resolved with a single additional maneuver. Notably, such rare occurrences do not compromise the overall efficacy of the CRP.

By contrast, the apogeotropic form of horizontal canal BPPV (hc-BPPV-apo) deserves particular attention. Here, otoconial debris may be either floating within the ampullary arm or adherent to the cupula [80], creating a more challenging clinical scenario—especially for less experienced clinicians. In our cohort, hc-BPPV-apo often required multiple maneuvers and longer treatment times, typically progressing through conversion to the geotropic form before final resolution. While most published series report this variant as less frequent than hc-BPPV-geo [81,82], in our population, the two occurred with equal incidence. This apparent discrepancy likely reflects the patient selection process, as our center receives a substantial proportion of referrals already evaluated elsewhere, including diagnostically complex cases [83].

From a diagnostic standpoint, the UBP was conclusive in 55 of 81 Group A patients (67.9%), rising to 87.5% when only hc-BPPV-geo and hc-BPPV-apo were considered. This confirms previous reports [61,62] and underscores the high sensitivity of the UBP in diagnosing horizontal canalolithiasis from the sitting position, thus avoiding supine testing and related symptoms. By contrast, diagnostic sensitivity for pc-BPPV was 59.65%. As previously reported, protocol sensitivity is strongly influenced by the time elapsed since symptom onset, being highest in patients assessed acutely in the emergency department and decreasing to below 70% after one week [63]. The low sensitivity of the UBP for pc-BPPV observed in the present study is therefore most likely related to patient referral patterns and selection bias.

Group A of our study included only the typical forms of BPPV, with a distribution that substantially reflected the traditional—though outdated—epidemiological scheme: posterior canal 80–90%, horizontal canal 10–20%, anterior canal 1–2% [84,85]. However, more than 40% of patients in our cohort fell into Group B, encompassing conditions not addressed by current guidelines. These atypical variants form a heterogeneous spectrum of vestibular disorders departing from the paradigms of typical BPPV and have been described individually over the years by various Authors. The terminology used here follows the original designations proposed in the literature—for example, “apogeotropic posterior semicircular canal BPPV” (apo-pc-BPPV) [29,31]—or descriptive criteria such as “sitting-up vertigo” (pc-BPPV-suv) [36]. Furthermore, the DBNy-BPPV-uc subgroup included seven patients (7/139, 5.04%) without a definite diagnosis, in whom a lithiasic origin was only hypothesized on the basis of clinical history, exclusion of other vestibular and neurological disorders, and the transient nature of symptoms; however, neither the affected side nor the specific canal involved could be determined, even retrospectively.

Most atypical forms shared a common denominator: positional nystagmus with a vertical vector, indicating involvement of the vertical canals. Most cases—namely the sum of patients with apo-pc-BPPV, ac-BPPV, and DBNy-BPPV-uc—presented with DBNy (38/58, 65.5%). DBNy was often accompanied by a torsional component that was more or less clearly defined. This torsional element is important for lateralization, yet it does not allow precise identification of the affected side and canal. For example, the torsional pattern of an apo-pc-BPPV on one side is identical to that of an ac-BPPV on the contralateral side [29,30,31,32]. Notably, recent evidence suggests that posterior canal involvement may be more frequent than anterior canal involvement in BPPV with DBNy during DHT [33,51]. The observation of a downbeat nystagmus with a concomitant torsional component during the UBP may therefore raise suspicion of apo-pc-BPPV, but cannot be regarded as conclusive for diagnostic purposes. When the UBP does not yield a conclusive diagnosis, it remains necessary to proceed with conventional positional tests in the supine position, as this represents the only reliable strategy for identifying atypical BPPV variants, despite the potential discomfort for the patient.

The detection of the affected canal in patients with non-paroxysmal DBNy may be challenging, as involvement of the non-ampullary arm of the posterior canal produces the same oculomotor responses as otoconia located in the contralateral anterior canal. Recent evidence suggests that DBNy may, at least in some cases, result from an incomplete CJ or ‘positional CJ,’ partially obstructing the canal lumen. Such an incomplete CJ would act as a low-pass filter within the affected canal, allowing the cupula to be activated by low-frequency stimuli (otoconial shifts) while preventing the ampullary receptor from responding to high-frequency inputs such as head impulses [51,52,53]. Previous studies have indeed demonstrated the utility of vHIT in detecting canal involvement in patients presenting with positional DBNy, reporting an overall sensitivity of 72.9% [51]. In our cohort, the peripheral origin of DBNy was clarified at bedside with video-Frenzel examination in 10 of 38 patients (26.3%), whereas in the remaining 28 patients, vHIT was performed, successfully identifying the affected canal in 16 cases (57.14%) through the detection of VOR gain values below the normal range in the corresponding canal.

A selectively reduced VOR gain was detected by vHIT in the two cases of complete CJ observed in this study. Both patients presented with persistent direction-fixed horizontal nystagmus and selective VOR-gain reduction in the HSC. CJ, first described by Epley [1], is attributed to particle impaction within the canal, with the fixed nystagmus explained by utriculofugal cupular displacement due to negative pressure between the clot and the cupula [1,86]. In both cases, repeated maneuvers resolved the CJ, converting the nystagmus into a direction-changing paroxysmal horizontal form at the sHYT, followed by normalization of vHIT.

Conversely, the diagnosis of pc-BPPV-cu and pc-BPPV-suv does not rely on instrumental tests but exclusively on the observation of changes in positional nystagmus induced by head movements.

Posterior canal cupulolithiasis (pc-BPPV-cu), first described by Epley, has long been considered rare; however, a recent study reported an incidence of 6.2% [87]. Cupulolithiasis is characterized by a non-paroxysmal nystagmus that persists as long as the provocative position is maintained, reaching maximum intensity when the affected cupula is aligned earth-horizontal [88,89]. In pc-BPPV-cu, vertigo and nystagmus are present in the DHT but are maximally elicited in the HHT. Diagnosis should be confirmed by the “inversion test,” in which the head is rotated 180° to the release position: if nystagmus ceases and then gradually reappears in the opposite direction, cupulolithiasis should be confirmed [1,19]. Although the diagnostic principles of posterior canal cupulolithiasis have been acknowledged by the Bárány Society guidelines [19], the underlying pathophysiology remains debated. The otoconial mass may adhere either to the canalar side or the utricular side of the cupula, with distinct therapeutic implications. Moreover, Oas [90] described a subtype of canalolithiasis in which debris is thought to float between the cupula, termed “short-arm posterior canal canalolithiasis,” which—according to his description and that of other authors [91]—shares some diagnostic features with cupulolithiasis. Adding further complexity, cases of concomitant long-arm cupulolithiasis and short-arm canalolithiasis of the posterior canal have also been reported [38,92]. In our series, irrespective of the proposed pathophysiological mechanism, we classified as pc-BPPV-cu all cases showing a non-paroxysmal, without latency, long-lasting up-beating nystagmus with an ipsilateral torsional component, elicited by the DHT, enhanced in the HHT, and associated with a positive inversion test.

Short-arm posterior canal canalolithiasis has also been suggested as the mechanism underlying sitting-up vertigo (pc-BPPV-suv) [48,93]. Scocco et al. [37] hypothesized that short-arm canalolithiasis may mimic the signs of a ‘heavy cupula’ during provocative testing (HHT and return to sitting after DHT). In this scenario, otoconia trapped on the utricular side of the ampulla may produce a sustained ampullofugal deflection in the provocative position. However, the inversion test would be negative, as otoconia would shift toward the ampullary opening or the vestibule, thereby relieving the stimulus on the cupula. Although several Authors have proposed short-arm canalolithiasis as the underlying mechanism of certain variants of lithiasic disorders, this concept has been the subject of considerable criticism. Anatomically, the utricular side of all three ampullae opens widely into the utricular cavity, and the cupula on its utricular surface is constantly exposed to otoconial debris. However, due to the broad communication between the ampulla and the utricle, a “plunger effect” on the utricular side is unlikely to occur, and consequently, no significant utricular deflection should be expected [16]. In a previous study, Scocco et al. [36] proposed an alternative mechanism, immune to such criticism. His original interpretation was based on the pericupular presence of otoconia located on the canal side, either aggregated into a dense mass or “trapped” due to a coexisting periampullary stenosis. In both scenarios, otoconial movement during the Dix–Hallpike maneuver (from the sitting to the supine head-hanging position) would be very limited. Upon returning to the sitting position, a slight ampullipetal shift in the otoconial mass would first occur, followed by a larger ampullifugal flow responsible for the characteristic nystagmus and vertigo observed upon sitting up.

Given the uncertain pathophysiology and the absence of established diagnostic criteria, although several maneuvers have been proposed for the treatment of atypical variants, no validated or evidence-based therapy currently exists for the atypical BPPV forms included in Group B. For this reason, in our series, multiple therapeutic strategies were combined, with the aim of mobilizing otoconial debris and converting the atypical form into a typical BPPV.

In our protocol, pc-BPPV-cu and pc-BPPV-suv were treated with a high-velocity maneuver in the plane of the affected posterior canal, under the hypothesis of cupulolithiasis or otoconial entrapment. The maneuver was designed to exploit linear acceleration and inertia to mobilize otoconia into the vestibule or, alternatively, to free them within the canal. This original procedure, termed the “Bascule maneuver,” was performed with the patient seated at the edge of the bed, legs dangling as in the Semont maneuver, and the head turned toward the healthy side. The patient was then rapidly moved first onto the healthy side with the nose facing downward and subsequently onto the affected side with the nose facing upward, each position maintained for ~20 s. These cycles were repeated until nystagmus either disappeared, changed direction, or tolerance was reached. A prospective study of this maneuver has recently been completed, although results are not yet published. Notably, in the two pc-BPPV-cu cases with immediate conversion observed during the same session, canaliths migrated into the horizontal canal, producing a conversion from pc-BPPV-cu to hc-BPPV-geo. This finding strongly supports the hypothesis of otoconial localization on the utricular side of the cupula.

Considering therapy, a key finding of our study is the marked difference between Group A and Group B in terms of clinical course and resolution. Group A patients (typical pc-BPPV and hc-BPPV) generally resolved rapidly with standard liberatory maneuvers—most commonly Epley and Gufoni—whereas Group B patients showed a more complex course, with significantly lower rates of immediate resolution and higher proportions requiring repeated or combined maneuvers.

This observation underscores a fundamental distinction between the two groups: whereas Group A largely reflects the classical clinical model of BPPV—most often corresponding to canalolithiasis, with free-floating debris easily displaced and high rates of single-visit resolution—Group B captures the heterogeneity of atypical variants, where anatomy, physiology, and comorbidities interact to reduce therapeutic efficacy.

Vestibular comorbidities were significantly more frequent in atypical canalolithiasis, consistent with prior literature [6,7]. Ménière’s disease [94,95], third mobile window syndrome [96,97], labyrinthine ischemic injury [98,99,100], and vestibular neuritis [101,102,103,104,105,106] may alter canal hydrodynamics and impair otoconial clearance, promoting debris accumulation in atypical sites or cupular adhesion. These mechanisms likely contribute to the complex nystagmus patterns and reduced therapeutic responsiveness observed in these patients. Clinically, recognition of this association is important, since recurrent or atypical BPPV should prompt consideration of a broader vestibular work-up [6].

In this scenario, the classification of multicanal BPPV (mc-BPPV) among atypical forms seems debatable, as it essentially represents the coexistence of multiple typical variants rather than a distinct pathophysiological entity. In our cohort, mc-BPPV accounted for 8.6% of cases, mostly presenting as bilateral pc-BPPV, as also reported in a previous series [107]. Bilateral involvement has often been associated with post-traumatic cases [27], whereas Shim et al. [26] in a large series reported that nearly 80% of mc-BPPV patients showed simultaneous involvement of two canals on the same side. Despite potentially complex nystagmus patterns during positional testing [108], therapeutic management remains relatively straightforward once each canal is correctly identified. Canalith repositioning maneuvers, applied sequentially or in combination, are usually effective: in our series, resolution was achieved within three visits in all cases, with 50% resolving after the first visit. Overall, mc-BPPV appears to behave more like an additive presentation of typical canalolithiasis than a real atypical variant.

This study has several limitations. Its retrospective single-center design may have introduced referral bias; however, although this may overestimate the incidence of atypical forms in primary referral centers, the observed diagnostic distribution appears consistent with that expected in higher-level centers. In addition, the relatively small sample size for some rare subgroups, the restriction of follow-up to four visits—potentially underestimating the persistence of particularly resistant cases—and the non-standardized therapeutic approach in Group B limit generalizability and reproducibility, but also highlight the challenges of managing BPPV variants for which therapeutic strategies remain to be defined.

Future studies should preferably be prospective and multicenter, with the aim of confirming the results emerging from this study and clarifying the pathophysiological contribution of vestibular comorbidities. Another crucial step is the development of standardized diagnostic and therapeutic protocols encompassing atypical variants. Advancing in these directions could help narrow the gap between guideline recommendations and everyday clinical practice, with a tangible impact on patient outcomes.

## 5. Conclusions

Atypical forms of BPPV proved more prevalent than previously estimated, accounting for nearly half of the cases in this cohort and reflecting the clinical complexity typically encountered in tertiary referral centers. These variants are relatively common, their pathophysiology is still debated, and they often show delayed therapeutic responses and greater resistance to repositioning maneuvers. Diagnosis relies on careful nystagmus analysis with video-Frenzel goggles, complemented when appropriate by instrumental testing such as vHIT.

Awareness of these variants is essential, as their recognition has direct implications for management and prognosis.

## Figures and Tables

**Table 1:** Typical and Atypical Forms of BPPV.

**Group A—Typical Forms**
Posterior canal canalolithiasis (pc-BPPV)
Horizontal canal BPPV, geotropic form (hc-BPPV-geo)
Horizontal canal BPPV, apogeotropic form (hc-BPPV-apo)
**Group B—Atypical Forms**
Anterior canal canalolithiasis (ac-BPPV)
Posterior canal cupulolithiasis (pc-BPPV-cu)
Multi-canal lithiasis (mc-BPPV)
Apogeotropic posterior canal canalolithiasis (apo-pc-BPPV)
Posterior canal BPPV with “sitting-up vertigo” (pc-BPPV-suv):
Canalith Jam
BPPV with positional downbeat nystagmus (unidentified canal) (DBNy-BPPV-uc)

Legend: BPPV, Benign Paroxysmal Positional Vertigo.

**Table 2:** Typical BPPV. Diagnostic Criteria.

Form	Maneuver	Findings
**PC canalolithiasis** ***(pc-BPPV)***	uHPT	*UBNy + ipsi torsion (±parox) in extension; reversal on forward bending*
	uLARP/RALP	*As above, limited to single plane (LARP/RALP)*
	SSPT	*Paroxysmal UBNy + ipsi torsion; reversal on sitting*
	DHT	*AS: paroxysmal UBNy + ipsi torsion; reversal on sitting*
**HC-BPPV,** **geotropic** ***(hc-BPPV-geo)***	uHPT	*Horizontal (±parox); ext → unaffected; flex → affected*
	uHRT	*Horizontal geotropic*
	SSPT	*Horizontal (±parox); beats toward unaffected side*
	sHYT	*Paroxysmal horizontal geotropic (both sides); stronger on affected side*
**HC-BPPV,** **apogeotropic** ***(hc-BPPV-apo)***	uHPT	*Horizontal; ext → affected; flex → unaffected*
	uHRT	*Horizontal apogeotropic*
	SSPT	*Horizontal (±parox); beats toward unaffected side*
	sHYT	*Horizontal apogeotropic (both sides; often non-paroxysmal); stronger on unaffected side*

Legend: AS, affected side; BPPV, Benign Paroxysmal Positional Vertigo; DHT, Dix–Hallpike Test; ext, head extension; flex, head flexion; HC, Horizontal (Lateral) Semicircular Canal; ipsi torsion, torsional component toward affected side; PC, Posterior Semicircular Canal; SSPT, sHYT, Supine Head Yaw Test; Seated-to-Supine Positioning Test; UBNy, Up-beating Nystagmus, uHPT, Upright Head Pitch Test; uHRT, Upright Head Roll Test; uLARP/RALP, Upright LARP/RALP Test; ±parox, often paroxysmal.

**Table 3:** Atypical BPPV. Diagnostic Criteria.

Form	Maneuver	Findings
**AC canalolithiasis** ***(ac-BPPV)***	uHPT	*DBNy; slight ipsi torsion ±*
	uLARP/RALP	*Similar findings, often on BS*
	DHT (AS/BS) + RP	*DBNy + torsion → AS; ±parox; transforms into typical form (pc-BPPV) or shows ↓ VOR gain at vHIT*
**PC cupulolithiasis** ***(pc-BPPV-cu)***	uHPT	*Non-parox UBNy+ ipsi torsion in ext*
	uLARP/RALP	*Similar findings on AS; may reverse on HS*
	DHT (AS)	*Non-parox UBNy + ipsi torsion*
	DHT (HS)	*DBNy + contra torsion;*
	HHT (AS)	*UBNy + ipsi torsion; > intense* vs. *DHT*
	INVERSION TEST	*Positive*
**Multi-canal lithiasis** ***(mc-BPPV)***	uHPT/uLARP/RALP/uHRT	*Ny compatible with canalolithiasis of >1 canal*
	DHT, sHYT	*Same findings*
**Apogeotropic** **PC canalolithiasis** ***(apo-pc-BPPV)***	uHPT	*DBNy + contra torsion*
	uLARP/RALP	*Similar findings, often on BS*
	DHT (AS), RP	*Non-parox DBNy; transforms into typical form (pc-BPPV), or shows ↓ VOR gain at vHIT*
**PC lithiasis** **with sitting-up vertigo *(pc-BPPV-suv):***	uHPT/uLARP/RALP	*Usually absent*
	Sitting up after DHT (AS) + RP	*UBNy + ipsi torsion*
	HHT	*UBNy + ipsi torsion*
	INVERSION TEST	*Negative*
**Canalith jam** ***(CJ)***	uHPT/uLARP/RALP/uHRT	*Direction-fixed, omnipositional horiz ny*
	sHYT (BS)	*Direction-fixed horiz ny; sometimes absent or weak reversion on one side; may convert → HC-BPPV; usually ↓ VOR gain at vHIT*
**BPPV with downbeat ny, unspecified canal** ***(DBNy-BPPV-uc)***	uHPT/uLARP/RALP	*DBNy ± ipsi torsion*
	DHT (BS), RP	*Non-parox DBNy ± torsion; no conversion to typical form; non-diagnostic vHIT; resolution ≤ 4 wks*

AS, affected side; AC, Anterior Semicircular Canal; BPPV, Benign Paroxysmal Positional Vertigo; BS, both sides; contra, contralateral; DBNy, Down-beating Nystagmus; DHT, Dix–Hallpike Test; ext, head extension; flex, head flexion; HC, Horizontal Semicircular Canal; HS, healthy side; HHT, Half–Dix–Hallpike Test; horiz, horizontal; ipsi, ipsilateral; ny, nystagmus; PC, Posterior Semicircular Canal; uHPT, Upright Head Pitch Test; uHRT, Upright Head Roll Test; sHYT, Supine Head Yaw Test; uLARP/RALP, Upright LARP/RALP Test; RP, Rose Position; UBNy, Up-beating Nystagmus; vHIT, Video Head Impulse Test; VOR, Vestibulo-Ocular Reflex; wks, weeks; ±parox, often paroxysmal; vert, vertical; ↓, reduced; >, more intense.

**Table 4:** The subgroup distribution. Group A, Typical BPPV; Group B, Atypical BPPV.

Group	Diagnosis	*n*	%
**Group A**	pc-BPPV	57	41
	hc-BPPV-geo	12	8.6
	hc-BPPV-apo	12	8.6
**Group B**	apo-pc-BPPV	25	18
	ac-BPPV	2	1.4
	DBNy-BPPV-uc	7	5
	pc-BPPV-cu	5	3.6
	pc-BPPV-suv	5	3.6
	CJ	2	1.4
	mc-BPPV	12	8.6

Legend: pc-BPPV, posterior canal canalolithiasis; hc-BPPV-geo, horizontal canal BPPV, geotropic form; hc-BPPV-apo, horizontal canal BPPV, apogeotropic form. apo-pc-BPPV, apogeotropic posterior canal canalolithiasis; mc-BPPV, multi-canal lithiasis; DBNy-BPPV-uc, BPPV with downbeat ny, unspecified canal; pc-BPPV-suv, posterior canal BPPV with sitting-up vertigo; pc-BPPV-cu, posterior canal cupulolithiasis; CJ, Canalith Jam; ac-BPPV, anterior canal canalolithiasis.

**Table 5:** Distribution of resolution paradigms in Group A and Group B.

Group	(a) Immediate Disappearance	(b) Conversion Same Visit	(c) Disappearance Next Visit	(d) Conversion Next Visit
**A**	60 (74.1%)	4 (4.9%)	14 (17.3%)	3 (3.7%)
**B**	15 (25.9%)	9 (15.5%)	27 (46.6%)	7 (12.1%)

## Data Availability

All data are presented in the article.

## Abbreviations

The following abbreviations are used in this manuscript:

Ac-BPPV	anterior canal canalolithiasis
Apo-pc-BPPV	apogeotropic posterior canal canalolithiasis
BPPV	benign paroxysmal positional vertigo
CJ	canalith jam
DBNy	down-beating nystagmus
DBNy-BPPV-uc	BPPV with down-beating nystagmus, unspecified canal
DHT	Dix–Hallpike Test
Hc-BPPV-apo	horizontal canal bppv, apogeotropic form
Hc-BPPV-geo	horizontal canal bppv, geotropic form
HHT	Half–Dix–Hallpike Test
Mc-BPPV	multi-canal lithiasis
Pc-BPPV	posterior canal canalolithiasis
Pc-BPPV-cu	posterior canal cupulolithiasis
Pc-BPPV-suv	posterior canal BPPV with sitting-up vertigo
RP	Rose Position
SHYT	Supine Head Yaw Test
SSPT	Seated-To-Supine Positioning Test
UHPT	Upright Head Pitch Test
UHRT	Upright Head Roll Test
ULARP/RALP	Upright Larp/Ralp Test
VHIT	Video Head Impulse Test
VOR	vestibulo-ocular reflex
